# Risk of Suboptimal Serum Sodium Correction According to Etiology in Severe Hyponatremia

**DOI:** 10.31662/jmaj.2026-0113

**Published:** 2026-08-14

**Authors:** Akira Nakamura, Takashin Nakayama, Tatsuhiko Azegami, Motoaki Komatsu, Kaori Hayashi

**Affiliations:** 1 Department of Internal Medicine, Keio University School of Medicine, Tokyo, Japan; 2 Department of Nephrology, Tokyo Saiseikai Central Hospital, Tokyo, Japan

**Keywords:** hyponatremia, etiology, correction rate, serum sodium, logistic regression

## Abstract

**Introduction:**

Achieving an appropriate serum sodium correction rate in hyponatremia is essential but remains challenging. Given that the rate of correction in hyponatremia may vary by underlying etiology, understanding this relationship could help optimize clinical management, but it remains insufficiently explored. To address this, we aimed to evaluate whether the risk of suboptimal correction varies across different etiological categories.

**Methods:**

This retrospective observational study included patients with severe hyponatremia (serum sodium level ≤120 mEq/L) at admission or during hospitalization at two tertiary care centers between January 2014 and June 2024. Patients were classified into nine etiological categories: kidney losses, non-kidney losses, syndrome of inappropriate antidiuresis (SIAD), adrenal insufficiency, low solute intake, polydipsia, heart failure, cirrhosis, and kidney disease. The association of etiological categories and the incidence of rapid (≥10 mEq/L) or slow (≤5 mEq/L) serum sodium correction within the initial 24 hours was assessed.

**Results:**

Among the 876 patients included, the median (interquartile range) age was 73 (62-82) years. The baseline serum sodium level was 118 (115-119) mEq/L, and the 24-hour correction rate was 4.8 (1.7-7.8) mEq/L. Logistic regression analysis revealed that, compared to SIAD, polydipsia (odds ratio [OR], 10.3; 95% confidence interval [CI], 4.81-22.0), adrenal insufficiency (OR, 5.32; 95% CI, 2.29-12.4), non-kidney losses (OR, 3.25; 95% CI, 1.38-7.66), and low solute intake (OR, 3.15; 95% CI, 1.73-5.76) were associated with rapid correction. Cirrhosis (OR, 2.24; 95% CI, 0.92-5.49), heart failure (OR, 1.79; 95% CI, 0.84-3.83), and kidney losses (OR, 1.48; 95% CI, 0.86-2.55) showed a tendency toward slower sodium correction.

**Conclusions:**

Correction rates varied by underlying etiology in severe hyponatremia. Etiological assessment may help predict correction rates and optimize management strategies in clinical practice.

## Introduction

Hyponatremia is the most frequently observed electrolyte disorder in clinical practice and affects approximately 20%-30% of hospitalized patients, resulting in increased morbidity and mortality ^(1), (2), (3)^. The rate of serum sodium correction is a critical factor in the management of hyponatremia ^(4), (5), (6)^. Increasing awareness of the risk of osmotic demyelination syndrome associated with overly rapid correction has led to a more cautious approach in recent years ^(7), (8), (9)^. Conversely, recent studies have highlighted that excessively slow correction may also be linked to poorer outcomes ^(9), (10)^. Taken together, these findings underscore the need to maintain an appropriate sodium correction rate within a narrow therapeutic window.

Hyponatremia reflects a disturbance of sodium–water homeostasis caused by diverse etiologies, including water excess, sodium loss, endocrine disorders, and organ dysfunction. Accurate identification of the underlying etiology may facilitate appropriate management, and various diagnostic approaches have been developed for this purpose ^(11), (12), (13)^. Although some previous studies have evaluated the impact of specific etiologies or volume status, comprehensive data covering a broad range of etiologies, particularly regarding their association with sodium correction rates, remain limited ^(4), (5), (6), (14), (15)^. Further investigation of these associations is an important research priority.

In this study, we aimed to clarify the relationship between underlying etiologies and 24-hour sodium correction patterns in severe hyponatremia.

## Materials and Methods

### Study population

This retrospective cohort study included patients who developed severe hyponatremia (serum sodium ≤120 mEq/L) either at admission or during hospitalization at Keio University Hospital or Tokyo Saiseikai Central Hospital between January 2014 and June 2024 ^(9), (16)^. Patients were excluded based on the following criteria: 1) obvious measurement errors; 2) age under 18 years; 3) blood glucose >500 mg/dL; 4) management without hospitalization; and 5) lack of follow-up sodium measurements within 48 hours. For patients with recurrent episodes of hyponatremia, only the first episode was included in the analysis. The Ethics Committee of Keio University School of Medicine reviewed and approved this study and all related protocols (approval number: 20241196). All procedures were conducted in compliance with the Declaration of Helsinki. Informed consent was obtained through an opt-out method via institutional websites.

### Data collection

Electronic medical records were reviewed to collect demographic and clinical information, including age, sex, height, weight, blood pressure, clinical setting at the onset of hyponatremia (inpatient or outpatient), care unit during the initial 24 hours (intensive care unit, high-acuity unit, or general ward), comorbidities, concurrent medications, and initial treatment (hypertonic saline administration or dialysis). The onset pattern of hyponatremia was classified as acute (≤48 hours), chronic (>48 hours), or uncertain when the time of onset was indeterminate. Laboratory parameters obtained at the time of hyponatremia onset included serum and urine concentrations of sodium and potassium, as well as chloride, albumin, total protein, uric acid, urea nitrogen, creatinine, thyroid-stimulating hormone, hemoglobin, cortisol, adrenocorticotropic hormone, and B-type natriuretic peptide (BNP). Serial serum sodium levels were collected throughout the hospital stay.

### Diagnostic approach

In this study, diagnoses were made using a standardized diagnostic approach ^(6), (13)^. Diagnoses were classified into nine etiological categories: 1) kidney losses; 2) non-kidney losses; 3) syndrome of inappropriate antidiuresis (SIAD); 4) adrenal insufficiency; 5) low solute intake; 6) polydipsia; 7) heart failure; 8) cirrhosis; and 9) kidney disease. Among the nine etiological categories, kidney losses, non-kidney losses, and kidney disease were characterized by especially heterogeneous pathophysiological backgrounds. Kidney losses referred to hypovolemic hyponatremia caused by renal sodium loss, including cases due to diuretics, cerebral salt wasting syndrome associated with intracranial lesions, and drug-induced renal salt-wasting nephropathy. Non-kidney losses referred to sodium loss of extrarenal origin, represented by conditions such as diarrhea and hemorrhage. Kidney disease referred to hypervolemic hyponatremia associated with acute kidney injury, chronic kidney disease, or nephrotic syndrome. As the causal relationship between hypothyroidism and hyponatremia remains controversial, hypothyroidism was not classified as an independent category in our analysis ^(4)^. Based on all available medical information before and after the development of hyponatremia, two expert nephrologists (selected from AN, TN, and MK) determined the underlying cause for each patient. In cases with multiple contributing factors, the primary etiology was used for analysis, and secondary causes were also documented. Discrepancies in diagnosis were resolved by consensus.

#### Calculation of sodium correction rate

The estimated serum sodium level at 24 hours (Na [24]) for each participant was calculated as follows: Na (24)= Na^a^ + [(Na^b^ - Na^a^) × (24 - T^a^) / (T^b^ - T^a^)], in which Na^a^ and T^a^ denote the closest serum sodium level and time point prior to 24 hours, and Na^b^ and T^b^ refer to the first values after the 24-hour mark, respectively ^(9), (16)^. The 24-hour change in serum sodium level was calculated as Na (24) – Na (0), where Na (0) represents the initial level at the onset of severe hyponatremia. To assess the impact on sodium correction rate, patients were stratified into three groups based on the 24-hour increase in serum sodium: ≤5, 5-10, and ≥10 mEq/L, defined as slow, optimal, and rapid correction, respectively ^(17), (18)^.

### Statistical analyses

Clinical characteristics and laboratory data were stratified into nine etiological categories. Continuous variables are reported as median values with interquartile ranges, and categorical variables are reported as counts and percentages. Based on the equation presented above, we calculated the 24-hour sodium correction rate for each etiological category and classified patients into three groups (slow, optimal, and rapid correction). For this calculation, patients who underwent dialysis within 24 hours were excluded, as dialysis alters serum sodium levels irrespective of etiology. To evaluate the association between etiology and the sodium correction rate, we performed logistic regression analyses to determine odds ratios (ORs) with 95% confidence intervals (CIs). Based on clinical reasoning and prior evidence, the following variables were selected as covariates in the multivariable model ^(4), (5), (9), (16)^: age, sex, high-acuity or intensive care unit stay, hypertonic saline administration, and baseline serum sodium level. To assess the robustness of our findings, additional sensitivity analyses were conducted with further adjustment for the Charlson comorbidity index, the clinical setting at the onset of hyponatremia, or onset patterns. We also performed analyses using alternative cutoff values for sodium correction (rapid ≥12 mEq/L and slow ≤6 mEq/L), based on guideline-recommended thresholds ^(4)^. All analyses were performed using STATA version 18.0 (StataCorp LLC, College Station, TX, USA).

## Results

### Characteristics and etiology of study patients

Of the 1,232 patients with serum sodium levels ≤120 mEq/L, 356 were excluded: 74 had obvious measurement errors, 24 were aged under 18 years, 19 had blood glucose levels >500 mg/dL, 126 were not hospitalized, and 113 had no follow-up measurement within 48 hours (Figure 1). Finally, 876 patients were included in the analysis.

**Figure 1.**
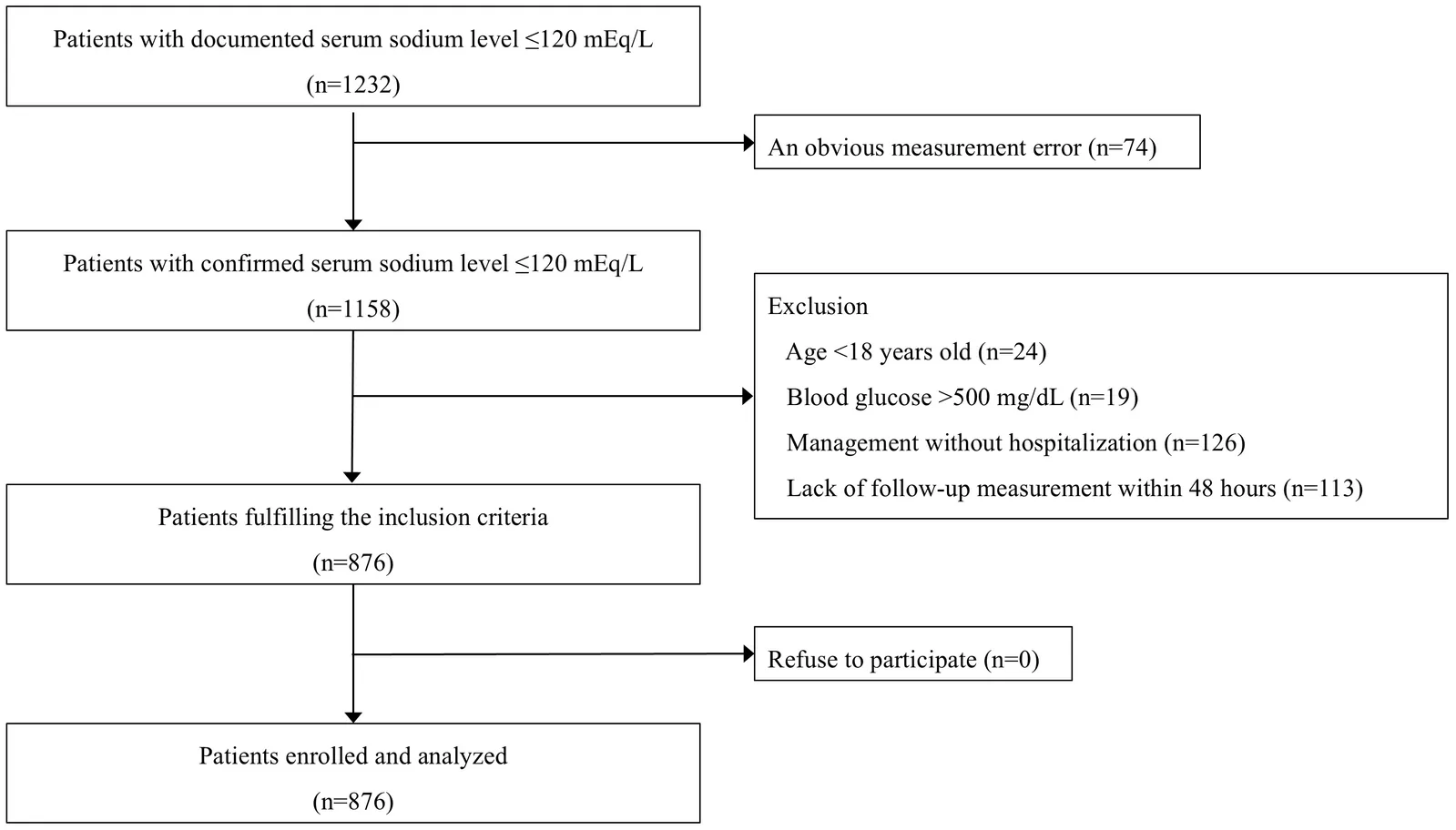
Flowchart of patient selection.

Baseline characteristics and laboratory data are described in Table 1 and Supplementary Table 1. The median (interquartile range [IQR]) age was 73 (62-82) years, 405 (46%) patients were female, and the baseline serum sodium level was 118 (115-119) mEq/L. According to the diagnostic algorithm, patients were classified into nine etiological categories: kidney losses (n = 94), non-kidney losses (n = 54), SIAD (n = 295), adrenal insufficiency (n = 38), low solute intake (n = 198), polydipsia (n = 58), heart failure (n = 56), cirrhosis (n = 49), and kidney disease (n = 34). Laboratory values for each etiological category are shown in Figure 2, with detailed data provided in Supplementary Table 1. Serum sodium was lowest in patients with polydipsia, whereas urine sodium was elevated in those with kidney losses, SIAD, and adrenal insufficiency. Uric acid was low in patients in the kidney losses and SIAD groups. BNP levels were elevated in patients in the heart failure and kidney disease groups. High urea nitrogen, low cortisol levels, and low albumin levels were specific to patients with kidney disease, adrenal insufficiency, and cirrhosis, respectively.

**Table 1.** Baseline Characteristics of Study Participants.

Characteristics		Total (n = 876)	Kidney losses (n = 94)	Non-kidney losses (n = 54)	SIAD (n = 295)	Adrenal insufficiency (n = 38)	Low solute intake (n = 198)	Polydipsia (n = 58)	Heart failure (n = 56)	Cirrhosis (n = 49)	Kidney disease (n = 34)
Age, years		73 (62-82)	75 (66-84)	73 (64-77)	74 (65-83)	64 (52-76)	75 (66-83)	61 (48-75)	76 (65-87)	56 (45-66)	77 (65-82)
Female		405 (46%)	44 (47%)	29 (54%)	122 (41%)	15 (39%)	105 (53%)	25 (43%)	37 (66%)	17 (35%)	11 (32%)
BMI (n = 802)		20 (18-23)	20 (17-22)	20 (18-23)	20 (17-22)	21 (19-23)	19 (17-22)	21 (19-25)	20 (17-24)	23 (20-25)	20 (19-22)
Systolic BP, mmHg		126 (109-144)	128 (112-143)	121 (100-136)	132 (118-149)	114 (103-133)	125 (107-142)	134 (117-150)	111 (90-132)	107 (95-123)	133 (115-148)
Diastolic BP, mmHg		73 (62-85)	74 (64-84)	69 (60-86)	77 (68-87)	69 (61-76)	73 (61-85)	80 (71-91)	63 (52-77)	62 (54-75)	68 (59-79)
Outpatient onset		448 (51%)	38 (40%)	31 (57%)	115 (39%)	21 (55%)	132 (67%)	53 (91%)	22 (39%)	22 (45%)	14 (41%)
Onset patterns											
	Acute	105 (12%)	20 (21%)	4 (7%)	46 (16%)	9 (24%)	17 (9%)	3 (5%)	3 (5%)	1 (2%)	2 (6%)
	Chronic	437 (50%)	44 (47%)	21 (39%)	148 (50%)	12 (32%)	90 (45%)	16 (28%)	40 (71%)	40 (82%)	26 (76%)
	Uncertain	334 (38%)	30 (32%)	29 (54%)	101 (34%)	17 (45%)	91 (46%)	39 (67%)	13 (23%)	8 (16%)	6 (18%)
Care units in the first 24 hours											
	General ward	764 (87%)	81 (86%)	45 (83%)	272 (92%)	35 (92%)	173 (87%)	41 (71%)	41 (73%)	43 (88%)	33 (97%)
	High-acuity unit	70 (8%)	10 (11%)	2 (4%)	16 (5%)	3 (8%)	17 (9%)	11 (19%)	9 (16%)	1 (2%)	1 (3%)
	Intensive care unit	42 (5%)	3 (3%)	7 (13%)	7 (2%)	0 (0%)	8 (4%)	6 (10%)	6 (11%)	5 (10%)	0 (0%)
Comorbidities											
	Alcohol use disorder	99 (11%)	6 (6%)	5 (9%)	13 (4%)	3 (8%)	25 (13%)	22 (38%)	2 (4%)	22 (45%)	1 (3%)
	CKD	147 (17%)	13 (14%)	9 (17%)	22 (7%)	3 (8%)	33 (17%)	6 (10%)	20 (36%)	9 (18%)	32 (94%)
	Chronic heart failure	212 (24%)	26 (28%)	6 (11%)	55 (19%)	0 (0%)	38 (19%)	13 (22%)	51 (91%)	2 (4%)	21 (62%)
	Cirrhosis	80 (9%)	1 (1%)	6 (11%)	9 (3%)	1 (3%)	7 (4%)	4 (7%)	1 (2%)	49 (100%)	3 (9%)
	Stroke	106 (12%)	10 (11%)	7 (13%)	46 (16%)	1 (3%)	25 (13%)	7 (12%)	5 (9%)	1 (2%)	4 (12%)
	Malignancy	380 (43%)	40 (43%)	23 (43%)	170 (58%)	21 (55%)	81 (41%)	8 (14%)	13 (23%)	17 (35%)	7 (21%)
	CCI	3 (1-6)	2 (1-4)	3 (1-6)	3 (1-6)	2 (0-6)	2 (1-5)	1 (0-3)	3 (2-5)	4 (3-5)	5 (3-7)
Medications before onset of hyponatremia											
	Loop diuretics	178 (20%)	17 (18%)	5 (9%)	35 (12%)	2 (5%)	33 (17%)	5 (9%)	39 (70%)	24 (49%)	18 (53%)
	Thiazide diuretics	62 (7%)	18 (19%)	2 (4%)	10 (3%)	0 (0%)	12 (6%)	5 (9%)	10 (18%)	1 (2%)	4 (12%)
	MRA	112 (13%)	15 (16%)	7 (13%)	16 (5%)	1 (3%)	23 (12%)	5 (9%)	21 (38%)	24 (49%)	0 (0%)
	Antiepileptic drugs	56 (6%)	9 (10%)	1 (2%)	22 (8%)	1 (3%)	15 (8%)	3 (5%)	2 (4%)	2 (4%)	1 (3%)
	Antipsychotics	50 (6%)	4 (4%)	2 (4%)	18 (6%)	0 (0%)	12 (6%)	10 (17%)	2 (4%)	0 (0%)	2 (6%)
	Antidepressants	60 (7%)	5 (5%)	7 (13%)	24 (8%)	0 (0%)	14 (7%)	7 (12%)	2 (4%)	0 (0%)	1 (3%)
	NSAIDs	128 (15%)	9 (10%)	5 (9%)	54 (18%)	12 (32%)	34 (17%)	8 (14%)	3 (5%)	3 (6%)	0 (0%)
	Opioids	96 (11%)	5 (5%)	6 (11%)	53 (18%)	2 (5%)	20 (10%)	2 (3%)	3 (5%)	3 (6%)	2 (6%)
Initial treatment after onset of hyponatremia											
	Hypertonic solution	257 (29%)	39 (41%)	11 (20%)	118 (40%)	18 (47%)	36 (18%)	21 (36%)	4 (7%)	7 (14%)	3 (9%)
	Dialysis	34 (4%)	0 (0%)	2 (4%)	2 (1%)	0 (0%)	2 (1%)	2 (3%)	2 (4%)	4 (8%)	20 (59%)

Abbreviations: BMI: body mass index; BP: blood pressure; CCI: Charlson comorbidity index; CKD: chronic kidney disease; MRA: mineralocorticoid receptor antagonist; NSAID: nonsteroidal anti-inflammatory drug; SIAD: syndrome of inappropriate antidiuresis.

**Figure 2.**
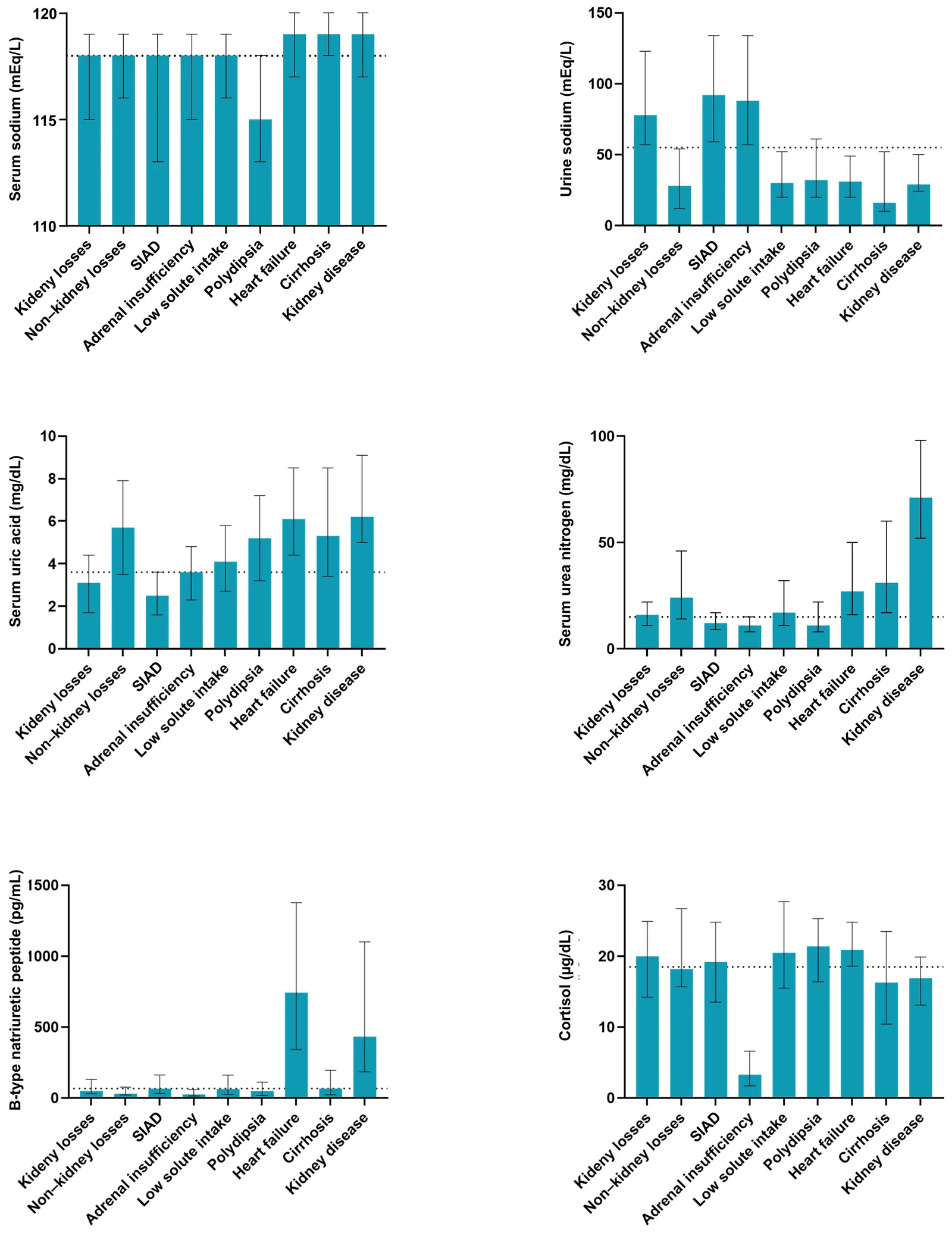
Laboratory data at the onset of hyponatremia. The dotted line indicates the median value for all categories. SIAD: syndrome of inappropriate antidiuresis.

### Sodium correction rate

After excluding 34 patients who underwent dialysis during the first 24 hours, the serum sodium correction rate was estimated using the specified equation. The overall median 24-hour serum sodium correction rate was 4.8 (IQR, 1.7-7.8) mEq/L. The results for each etiological category are illustrated in Figure 3. Among these, patients in the polydipsia group exhibited the highest correction rate at 11.1 (6.9-15.4) mEq/L, followed by the adrenal insufficiency group at 8.3 (2.2-10.6) mEq/L, the non-kidney losses group at 7.1 (5.0-9.6) mEq/L, and the low solute intake group at 5.7 (4.0-8.8) mEq/L. Patients in the SIAD group had a correction rate of 3.6 (1.0-6.3) mEq/L, while those in the kidney losses, heart failure, and kidney disease groups showed lower rates of 2.9 (−0.1 to 5.7) mEq/L, 2.8 (0.1-4.7) mEq/L, and 2.5 (0.8-6.0) mEq/L, respectively. Patients in the cirrhosis group had the lowest correction rate at 1.0 (−0.5 to 4.2) mEq/L. Overall, 453 (54%) patients experienced slow, 256 (30%) experienced optimal correction, and 133 (16%) experienced rapid sodium correction; further details by etiology are provided in Supplementary Table 2.

**Figure 3.**
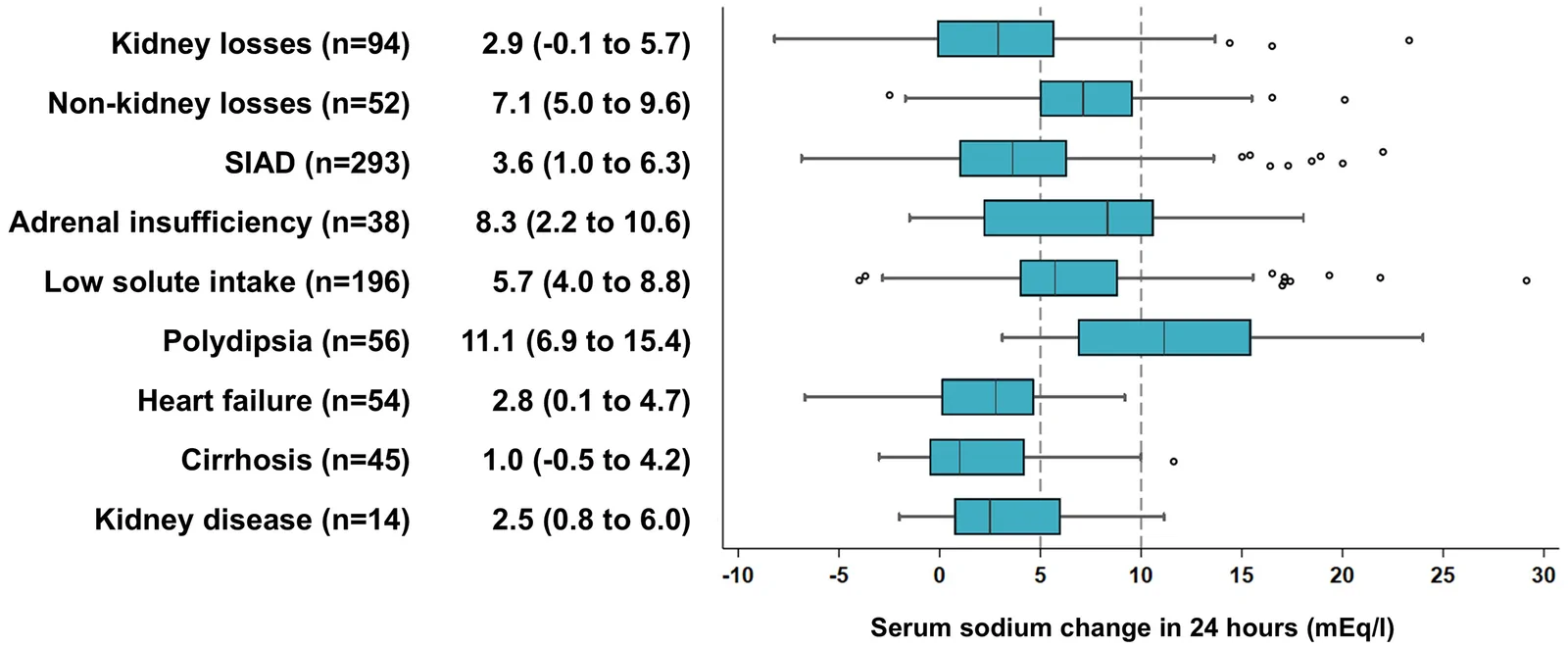
Change in serum sodium level over the first 24 hours. The dotted lines indicate 5 and 10 mEq/L. SIAD: syndrome of inappropriate antidiuresis.

### Logistic regression analyses

Logistic regression analyses were performed to determine the association between etiological category and rapid correction, using SIAD as the reference group because it included the largest number of patients (Table 2). In the fully adjusted model accounting for clinical characteristics and baseline serum sodium level, polydipsia, adrenal insufficiency, non-kidney losses, and low solute intake were most strongly associated with rapid correction, with ORs of 10.3 (95% CI, 4.81-22.0), 5.32 (95% CI, 2.29-12.4), 3.25 (95% CI, 1.38-7.66), and 3.15 (95% CI, 1.73-5.76), respectively. Furthermore, in the multivariable analysis assessing the association with slow correction, patients with cirrhosis, heart failure, and kidney losses showed an increased risk, although these associations did not reach statistical significance (adjusted ORs: 2.24 [95% CI, 0.92-5.49], 1.79 [95% CI, 0.84-3.83], and 1.48 [95% CI, 0.86-2.55], respectively) (Table 3). Sensitivity analyses additionally adjusted for the Charlson comorbidity index, the clinical setting at hyponatremia onset, or onset patterns showed consistent results (Supplementary Tables 3 and 4). Sensitivity analyses using alternative cutoff values also yielded similar results (Supplementary Tables 5 and 6).

**Table 2.** Associations of Etiology with Rapid Correction in Hyponatremia.

	Crude	Model 1	Model 2
Kidney losses	1.22 (0.57-2.64)	1.26 (0.58-2.75)	1.10 (0.47-2.55)
Non-kidney losses	3.08 (1.44-6.59)	2.77 (1.27-6.02)	3.25 (1.38-7.66)
SIAD	1 [reference]	1 [reference]	1 [reference]
Adrenal insufficiency	5.34 (2.44-11.7)	4.45 (2.00-9.94)	5.32 (2.29-12.4)
Low solute intake	2.47 (1.44-4.22)	2.44 (1.41-4.22)	3.15 (1.73-5.76)
Polydipsia	12.7 (6.56-24.7)	10.5 (5.28-20.8)	10.3 (4.81-22.0)
Heart failure *	-	-	-
Cirrhosis	0.48 (0.11-2.09)	0.33 (0.07-1.47)	0.57 (0.12-2.67)
Kidney disease	0.79 (0.10-6.28)	0.75 (0.09-6.12)	1.57 (0.19-12.9)

Crude and adjusted odds ratios (with 95% confidence intervals) associated with rapid correction in patients with hyponatremia are shown. Model 1 accounts for age and sex. Model 2 accounts for age, sex, high-acuity or intensive care unit stay, hypertonic solution administration, and baseline serum sodium level. Abbreviation: SIAD: syndrome of inappropriate antidiuresis. *No incidence of overcorrection in hyponatremia due to heart failure.

**Table 3.** Associations of Etiology with Slow Correction in Hyponatremia.

	Crude	Model 1	Model 2
Kidney losses	1.34 (0.81-2.21)	1.35 (0.82-2.24)	1.48 (0.86-2.55)
Non-kidney losses	0.23 (0.12-0.44)	0.24 (0.13-0.46)	0.19 (0.09-0.37)
SIAD	1 [reference]	1 [reference]	1 [reference]
Adrenal insufficiency	0.29 (0.14-0.60)	0.31 (0.15-0.64)	0.28 (0.13-0.60)
Low solute intake	0.37 (0.25-0.53)	0.38 (0.26-0.55)	0.28 (0.19-0.42)
Polydipsia	0.07 (0.03-0.16)	0.07 (0.03-0.18)	0.07 (0.03-0.18)
Heart failure	1.98 (1.00-3.93)	2.15 (1.08-4.29)	1.79 (0.84-3.83)
Cirrhosis	3.08 (1.33-7.13)	3.45 (1.47-8.11)	2.24 (0.92-5.49)
Kidney disease	1.02 (0.33-3.12)	1.03 (0.33-3.16)	0.63 (0.20-1.98)

Crude and adjusted odds ratios (with 95% confidence intervals) associated with slow correction in patients with hyponatremia are shown. Model 1 accounts for age and sex. Model 2 accounts for age, sex, high-acuity or intensive care unit stay, hypertonic solution administration, and baseline serum sodium level. Abbreviation: SIAD: syndrome of inappropriate antidiuresis.

## Discussion

This study demonstrates that the rate of serum sodium correction differs substantially depending on the underlying etiology of severe hyponatremia. Logistic regression analyses showed that polydipsia, adrenal insufficiency, non-kidney losses, and low solute intake were associated with rapid correction, whereas cirrhosis, heart failure, and kidney losses were linked to slow correction.

Although achieving an appropriate rate of serum sodium concentration is essential in the management of hyponatremia, it remains a considerable clinical challenge. Considering the risk of osmotic demyelination syndrome, international guidelines recommend that sodium correction should not exceed 8-12 mEq/L within 24 hours ^(4), (5)^. As a result, the magnitude of correction has indeed become substantially more conservative over the past few decades ^(9)^. However, several recent studies have reported an association between overly slow correction and adverse outcomes, highlighting the need to avoid slow correction ^(9), (10)^. The optimal range for sodium correction rate is narrow, making it difficult to attain in clinical settings. Formulas derived from the Edelman equation are commonly used to predict sodium correction rates, although their accuracy remains limited in real-world settings ^(19), (20), (21), (22)^. In this context, consideration of the underlying pathophysiology of hyponatremia may provide a more practical approach. In a study of 13 patients with polydipsia-related hyponatremia, Cheng et al. ^(14)^ demonstrated a high incidence of rapid sodium correction. Chung et al. ^(23)^, through volume status assessment in 58 patients, demonstrated an association between hypovolemic status and accelerated sodium correction. Similarly, using data from the Hyponatremia Registry, Cukoski et al. ^(15)^ also revealed that hypovolemic and thiazide-associated hyponatremia were linked to more pronounced sodium correction. However, comprehensive investigations exploring the relationship between etiology and correction rate, beyond volume status or specific causes, remain limited.

To our knowledge, this is the first study to systematically evaluate the clinical features associated with detailed etiological categories of hyponatremia. The underlying causes were classified by two board-certified nephrologists based on a comprehensive review of electronic medical records, including clinical course, physical findings, laboratory data, and imaging studies. As expected, patients with adrenal insufficiency exhibited low cortisol levels, and those with heart failure or kidney disease had elevated BNP levels, supporting the accuracy and validity of our etiological classification. Urinary sodium tended to be high not only in patients with kidney losses and SIAD but also in those with adrenal insufficiency. Baseline sodium levels were lowest in patients with polydipsia. These findings are generally consistent with existing literature ^(20), (24)^; however, notable overlap between etiologies was observed, with 47.9% of cases involving possible secondary contributors. While algorithm-based approaches are certainly useful, hyponatremia often arises from multifactorial mechanisms, underscoring the need for flexible clinical judgment.

The association between etiological categories and rapid correction was assessed using multivariate logistic regression. Compared with SIAD, the most common etiology, polydipsia, adrenal insufficiency, non-kidney losses, and low solute intake were significantly associated with a higher risk of rapid correction. Although the exact mechanism remains unclear, patients with polydipsia typically consume several liters of free water daily. Upon hospitalization, fluid restriction may induce a rapid shift in water balance that exceeds the kidney’s adaptive capacity, thereby increasing the risk of rapid correction. Suspected excessive polydipsia may necessitate close monitoring of urine output and early consideration of free water replacement. Adrenal insufficiency induces hyponatremia through both an insufficient aldosterone effect and increased antidiuretic hormone (ADH) secretion. Steroid therapy suppresses ADH and promotes water diuresis, potentially leading to rapid sodium correction. Therefore, careful observation following steroid administration is essential. Patients with adrenal insufficiency may also show SIAD-like laboratory profiles (excluding cortisol) and might be candidates for hypertonic saline; however, the concurrent use of steroids and hypertonic saline appears to be high-risk and warrants meticulous consideration. When hyponatremia is precipitated by non-kidney losses or low solute intake, initial intravascular volume restoration may trigger an abrupt decline in ADH secretion, with the attendant risk of rapid sodium repletion. In these scenarios, prudent clinical attention is therefore paramount.

Conditions such as cirrhosis, heart failure, and kidney losses showed a tendency toward slower sodium correction. In states of fluid overload, such as cirrhosis and heart failure, impaired water excretion makes sodium correction challenging ^(25), (26)^. Moreover, the use of hypertonic saline is generally avoided due to concerns about exacerbating volume overload. However, to avoid excessively slow correction, a multidisciplinary approach, including the combined use of various diuretics as appropriate and active adjustment of oral salt and fluid intake, is desirable ^(27)^. A substantial proportion of patients with slow correction in the kidney loss group had chemotherapy-induced salt-wasting nephropathy or cerebral salt wasting secondary to intracranial disease or surgery ^(28), (29)^. These conditions may be accompanied by persistent natriuresis, for which hypertonic saline represents a reasonable therapeutic option.

Several limitations of this study should be noted. Despite extensive adjustment for potential confounders, the retrospective nature of the study may have left residual biases unaddressed. The lack of data on symptom severity, a key factor in clinical management, represents an important unmeasured clinical factor influencing sodium correction and may affect the interpretation of our findings. Furthermore, differences in treatment strategies across etiological categories may have influenced the observed sodium correction patterns, as some therapeutic interventions (e.g., fluid management or etiology-directed therapies) are determined by the underlying etiology and may lie on the causal pathway between etiology and sodium correction rate. Accordingly, this study was designed to evaluate associations rather than causal effects, and the findings should be interpreted in that context. The etiological classification, while conducted by two board-certified nephrologists, remains subject to some diagnostic uncertainty. Nonetheless, the associated biochemical profiles were largely consistent with existing knowledge. Because the etiology of hyponatremia is not always immediately apparent at presentation and the initial etiological assessment may differ from the final diagnosis, these findings should be interpreted carefully in clinical settings. Nevertheless, early clinical features and preliminary assessments often provide important clues, and understanding the general tendencies associated with different etiologies may help anticipate sodium correction patterns and inform early monitoring strategies. In addition, while we applied widely used formulas to estimate sodium correction rate, their precision in real-world settings remains to be further substantiated. Lastly, as the study population was derived from tertiary care institutions and included patients with significant comorbidities, caution is warranted in extrapolating these findings to broader patient populations.

Our analyses revealed that correction rates varied depending on the underlying cause of severe hyponatremia. In comparison to SIAD, polydipsia, adrenal insufficiency, non-kidney losses, and low solute intake were associated with a higher risk of rapid correction, whereas cirrhosis, heart failure, and kidney losses showed a greater tendency toward slow correction. These findings suggest that careful assessment of the underlying etiology may facilitate the safe and effective management of hyponatremia.

## Article Information

### Author Contributions

Research idea and study design: Akira Nakamura, Takashin Nakayama, Tatsuhiko Azegami; data acquisition: Akira Nakamura, Takashin Nakayama, Motoaki Komatsu; data analysis/interpretation: Akira Nakamura, Takashin Nakayama, Tatsuhiko Azegami; statistical analysis: Akira Nakamura, Takashin Nakayama, Tatsuhiko Azegami; supervision or mentorship: Kaori Hayashi.

### Conflicts of Interest

None

### IRB Approval Code and Name of the Institution

The Ethics Committee of Keio University School of Medicine reviewed and approved this study and all related protocols (approval number: 20241196).

## Supplement

Supplementary Material
