# Supplementary material for: Risk of Suboptimal Serum Sodium Correction According to Etiology in Severe Hyponatremia
Source: JMA J. 2026 Aug 14;9(5):1287–95. doi: 10.31662/jmaj.2026-0113 (PMC13639670; doi:10.31662/jmaj.2026-0113)
Supplement: Supplementary file 1 [file 2433-3298-9-5-1287-s001.pdf]

Supplemental Materials

Risk of Suboptimal Serum Sodium Correction According to Etiology in Severe Hyponatremia

Authors:

Akira Nakamura, Takashin Nakayama, Tatsuhiko Azegami, Motoaki Komatsu, Kaori Hayashi

Contents:

Supplemental Table 1. Laboratory Data of Study Patients

Supplemental Table 2. Distribution of Sodium Correction Rates: Slow, Optimal, and Rapid Correction

Supplemental Table 3. Associations of Etiology with Rapid Correction in Hyponatremia (Sensitivity Analysis with Additional Covariates)

Supplemental Table 4. Associations of Etiology with Slow Correction in Hyponatremia (Sensitivity Analysis with Additional Covariates)

Supplemental Table 1. Laboratory Data of Study Patients

	Total (n = 876)	Kidney losses (n = 94)	Non-kidney losses (n = 54)	SIAD (n = 295)	Adrenal insufficiency (n = 38)	Low solute intake (n = 198)	Polydipsia (n = 58)	Heart failure (n = 56)	Cirrhosis (n = 49)	Kidney disease (n = 34)
Sodium, mEq/L (n = 876)	118 (115-119)	118 (116-119)	118 (113-119)	118 (115-119)	118 (115-119)	118 (116-119)	115 (113-118)	119 (117-120)	119 (118-120)	119 (117-120)
Potassium, mEq/L (n = 876)	4.4 (3.9-5.0)	4.5 (3.7-5.0)	4.6 (4.1-5.2)	4.4 (3.9-4.9)	4.3 (4.0-4.8)	4.4 (3.9-5.0)	3.9 (3.6-4.8)	4.6 (4.3-5.2)	4.9 (4.3-5.2)	4.4 (4.0-5.0)
Chloride, mEq/L (n = 869)	83 (80-86)	83 (80-85)	83 (80-87)	83 (80-86)	84 (81-86)	83 (80-86)	81 (76-84)	84 (80-87)	86 (83-88)	84 (80-88)
Albumin, g/dL (n = 849)	3.2 (2.7-3.8)	3.6 (3.2-4.0)	3.0 (2.4-4.0)	3.2 (2.6-3.7)	3.5 (3.0-3.9)	3.2 (2.6-3.8)	3.9 (3.6-4.2)	3.0 (2.7-3.2)	2.5 (2.1-2.8)	2.8 (2.2-3.4)
Total protein, mg/dL (n = 843)	6.3 (5.6-6.9)	6.5 (6.0-7.1)	6.2 (5.2-7.0)	6.3 (5.6-6.8)	6.4 (6.0-6.8)	6.2 (5.5-6.8)	6.9 (6.3-7.2)	6.4 (5.6-6.9)	5.8 (5.2-6.4)	5.8 (5.4-6.4)
Uric acid, mg/dL (n = 809)	3.6 (2.2-5.6)	3.1 (1.7-4.4)	5.7 (3.5-7.9)	2.5 (1.6-3.6)	3.6 (2.3-4.8)	4.1 (2.7-5.8)	5.2 (3.2-7.2)	6.1 (4.4-8.5)	5.3 (3.4-8.5)	6.2 (5.0-9.1)
Urea nitrogen, mg/dL (n = 876)	15 (10-27)	16 (11-22)	24 (14-46)	12 (9-17)	11 (8-15)	17 (11-32)	11 (8-22)	27 (16-50)	31 (17-60)	71 (52-98)
Creatinine, mg/dL (n = 875)	0.7 (0.5-1.1)	0.7 (0.5-1.0)	0.9 (0.6-1.9)	0.6 (0.4-0.8)	0.6 (0.5-0.9)	0.8 (0.5-1.3)	0.7 (0.5-1.1)	1.0 (0.6-1.8)	1.1 (0.8-2.2)	6.5 (4.6-8.5)
Hemoglobin, g/dL (n = 876)	11.1 (9.6-12.6)	12.0 (10.3-13.2)	10.7 (8.4-12.4)	11.2 (9.8-12.5)	11.9 (10.1-12.8)	10.9 (9.3-12.5)	12.2 (10.3-13.7)	11.0 (9.4-12.5)	10.6 (8.9-11.9)	9.6 (8.0-11.0)
Cortisol, μg/dL (n = 447)	18.5 (13.0-24.9)	20.0 (14.2-24.9)	18.2 (15.7-26.7)	19.2 (13.5-24.8)	3.3 (1.7-6.6)	20.5 (15.5-27.7)	21.4 (16.4-25.3)	20.9 (18.6-24.8)	16.3 (10.4-23.5)	16.9 (13.1-19.9)
ACTH, pg/mL (n = 397)	24.1 (13.9-40.2)	30.7 (17.8-45.3)	21.8 (14.3-39.5)	26.6 (14.8-41.4)	14.6 (2.9-34.7)	22.1 (15.9-37.6)	23.4 (7.9-38.6)	20.7 (13.3-31.4)	19.9 (9.4-31)	41.1 (30.3-47.4)
TSH, μU/mL (n = 583)	1.8 (0.9-3.2)	1.6 (0.8-3.1)	1.3 (0.7-3.2)	2.1 (1.0-3.3)	1.9 (0.5-4.7)	1.5 (0.7-2.7)	1.5 (0.7-2.5)	2.2 (1.4-4.9)	3.0 (2.0-5.1)	1.8 (1.2-2.9)
BNP, pg/mL (n = 524)	67 (29-205)	50 (30-132)	30 (21-77)	65 (31-163)	25 (14-59)	63 (25-161)	49 (17-112)	743 (342-1378)	66 (23-195)	432 (185-1101)
Urine sodium, mEq/L (n = 584)	55 (27-99)	78 (57-123)	28 (12-54)	92 (59-134)	88 (57-134)	30 (20-52)	32 (20-61)	31 (20-49)	16 (10-52)	29 (24-50)
Urine potassium, mEq/L (n = 569)	30 (20-43)	29 (19-44)	30 (20-47)	34 (25-47)	34 (24-43)	30 (17-42)	22 (11-34)	31 (13-44)	30 (22-39)	14 (12-30)

Abbreviations: SIAD, syndrome of inappropriate antidiuresis; ACTH, adrenocorticotropic hormone; TSH, thyroid-stimulating hormone; BNP, B-type natriuretic peptide.

Supplemental Table 2. Distribution of Sodium Correction Rates: Slow, Optimal, and Rapid Correction

	Total (n = 842)	Kidney losses (n = 94)	Non-kidney losses (n = 52)	SIAD (n = 293)	Adrenal insufficiency (n = 38)	Low solute intake (n = 196)	Polydipsia (n = 56)	Heart failure (n = 54)	Cirrhosis (n = 45)	Kidney disease (n = 14)
Slow correction	453 (54%)	66 (70%)	15 (29%)	187 (64%)	13 (34%)	77 (39%)	6 (11%)	42 (78%)	38 (84%)	9 (64%)
Optimal correction	256 (30%)	18 (19%)	25 (48%)	80 (27%)	12 (32%)	81 (41%)	19 (34%)	12 (22%)	5 (11%)	4 (29%)
Rapid correction	133 (16%)	10 (11%)	12 (23%)	26 (9%)	13 (34%)	38 (19%)	31 (55%)	0 (0%)	2 (4%)	1 (7%)

Abbreviation: SIAD, syndrome of inappropriate antidiuresis.

Supplemental Table 3. Associations of Etiology with Rapid Correction in Hyponatremia (Sensitivity Analysis with Additional Covariates)

	Crude	Model 2	Model 3	Model 4
Kidney losses	1.22 (0.57-2.64)	1.10 (0.47-2.55)	1.01 (0.43-2.38)	1.08 (0.46-2.51)
Non-kidney losses	3.08 (1.44-6.59)	3.25 (1.38-7.66)	3.34 (1.41-7.90)	3.35 (1.41-7.91)
SAID	1 [Reference]	1 [Reference]	1 [Reference]	1 [Reference]
Adrenal insufficiency	5.34 (2.44-11.7)	5.32 (2.29-12.4)	5.36 (2.29-12.5)	5.43 (2.33-12.7)
Low solute intake	2.47 (1.44-4.22)	3.15 (1.73-5.76)	3.27 (1.78-6.01)	3.29 (1.78-6.08)
Polydipsia	12.7 (6.56-24.7)	10.3 (4.81-22.0)	8.93 (4.14-19.2)	11.1 (5.02-24.4)
Heart failure *	-	-	-	-
Cirrhosis	0.48 (0.11-2.09)	0.57 (0.12-2.67)	0.71 (0.15-3.39)	0.58 (0.12-2.70)
Kidney disease	0.79 (0.10-6.28)	1.57 (0.19-12.9)	1.92 (0.23-15.8)	1.59 (0.19-13.0)

Crude and adjusted odds ratios (with 95% confidence intervals) associated with rapid correction in patients with hyponatremia are shown. Model 2 accounts for age, sex, high-acuity or intensive care unit stay, hypertonic solution administration, and baseline serum sodium level. Model 3 accounts for age, sex, high-acuity or intensive care unit stay, hypertonic solution administration, baseline serum sodium level, and Charlson comorbidity index. Model 4 accounts for age, sex, high-acuity or intensive care unit stay, hypertonic solution administration, baseline serum sodium level, and setting at hyponatremia onset. Abbreviation: SIAD, syndrome of inappropriate antidiuresis. *No incidence of overcorrection in hyponatremia due to heart failure.

Supplemental Table 4. Associations of Etiology with Slow Correction in Hyponatremia (Sensitivity Analysis with Additional Covariates)

	Crude	Model 2	Model 3	Model 4
Kidney losses	1.34 (0.81-2.21)	1.48 (0.86-2.55)	1.54 (0.89-2.66)	1.47 (0.86-2.54)
Non-kidney losses	0.23 (0.12-0.44)	0.19 (0.09-0.37)	0.18 (0.09-0.37)	0.19 (0.10-0.39)
SIAD	1 [Reference]	1 [Reference]	1 [Reference]	1 [Reference]
Adrenal insufficiency	0.29 (0.14-0.60)	0.28 (0.13-0.60)	0.29 (0.13-0.61)	0.31 (0.14-0.66)
Low solute intake	0.37 (0.25-0.53)	0.28 (0.19-0.42)	0.28 (0.19-0.43)	0.32 (0.21-0.48)
Polydipsia	0.07 (0.03-0.16)	0.07 (0.03-0.18)	0.08 (0.03-0.20)	0.10 (0.04-0.24)
Heart failure	1.98 (1.00-3.93)	1.79 (0.84-3.83)	1.73 (0.81-3.70)	1.78 (0.83-3.85)
Cirrhosis	3.08 (1.33-7.13)	2.24 (0.92-5.49)	2.03 (0.82-5.03)	2.43 (0.99-6.00)
Kidney disease	1.02 (0.33-3.12)	0.63 (0.20-1.98)	0.59 (0.19-1.86)	0.65 (0.21-2.07)

Crude and adjusted odds ratios (with 95% confidence intervals) associated with slow correction in patients with hyponatremia are shown. Model 2 accounts for age, sex, high-acuity or intensive care unit stay, hypertonic solution administration, and baseline serum sodium level. Model 3 accounts for age, sex, high-acuity or intensive care unit stay, hypertonic solution administration, baseline serum sodium level, and Charlson comorbidity index. Model 4 accounts for age, sex, high-acuity or intensive care unit stay, hypertonic solution administration, baseline serum sodium level, and setting at hyponatremia onset. Abbreviation: SIAD, syndrome of inappropriate antidiuresis.
